# Case report: Cryoablation as a novel bridging strategy prior to CAR-T cell therapy for B cell malignancies with bulky disease

**DOI:** 10.3389/fonc.2023.1008828

**Published:** 2023-01-27

**Authors:** Xiaomin Zhang, Jinming Wu, Liangliang Qiao, Lixuan Chen, Chaolin Chen, Hui Zhang, Rongcheng Luo, Yang Xiao

**Affiliations:** ^1^ Department of Hematology, Jinshazhou Hospital of Guangzhou University of Chinese Medicine, Guangzhou, China; ^2^ Institute of Clinical Medicine College, Guangzhou University of Chinese Medicine, Guangzhou, China; ^3^ Translational Medicine Center, The Second Affiliated Hospital of Guangzhou Medical University, Guangzhou, China; ^4^ School of Medicine, Jishou University, Jishou, China; ^5^ Thoracic Surgery, Jinshazhou Hospital of Guangzhou University of Chinese Medicine, Guangzhou, China; ^6^ Department of Hematology, Shenzhen Qianhai Shekou Pilot Free Trade Zone Hospital, Shenzhen, China

**Keywords:** CAR-T cell therapy, cryoablation, multiple myeloma, primary cutaneous diffuse large B-cell lymphoma leg type, bridging therapy

## Abstract

Chimeric antigen receptor (CAR) T-cell therapy has emerged as a powerful immunotherapy in relapsed/refractory (R/R) hematological malignancies, especially in R/R B-cell acute lymphocytic leukemia (B-ALL), non-Hodgkin lymphoma (NHL), and multiple myeloma (MM). To prevent disease progression and reduce tumor burden during CAR-T cell manufacturing, bridging therapies prior to CAR-T cell infusion are crucial. At present, it has been demonstrated that targeted therapy, radiotherapy and autologous stem cell transplantation (ASCT) could serve as effective bridging strategies. However, whether cryoablation could serve as a novel bridging strategy is unknown. In this paper, we report 2 cases of R/R B cell malignancies with bulky disease that were successfully treated with a combination of cryoablation and CAR-T cell therapy. Patient 1 was a 65-year-old female who was diagnosed with R/R MM with extramedullary disease (EMD). She was enrolled in the anti-BCMA CAR-T cell clinical trial. Patient 2 was a 70-year-old man who presented with a subcutaneous mass in the right anterior thigh and was diagnosed with primary cutaneous diffuse large B cell lymphoma, leg type (PCLBCL-LT) 1 year ago. He failed multiline chemotherapies as well as radiotherapy. Thus, he requested anti-CD19 CAR-T cell therapy. Unfortunately, they all experienced local progression during CAR-T cell manufacturing. To rapidly achieve local tumor control and reduce tumor burden, they both received cryoablation as a bridging therapy. Patient 1 achieved a very good partial response (VGPR) 1 month after CAR-T cell infusion, and patient 2 achieved a partial response (PR) 1 month after CAR-T cell infusion. In addition, adverse effects were tolerable and manageable. Our study demonstrated the favorable safety and efficacy of combination therapy with cryoablation and CAR-T cell therapy for the first time, and it also indicates that cryoablation could serve as a novel therapeutic strategy for local tumor control in B cell malignancies.

## 1 Introduction

Chimeric antigen receptor T (CAR-T) cell therapy is a promising adoptive T cell therapy, in which T cells are collected from peripheral blood and then genetically engineered to express CAR molecules that can specifically recognize tumor antigens without antigen processing and presentation. It represents a major breakthrough in cancer immunotherapy, which has achieved unprecedented success in B cell malignancies in recent years, especially in B-cell acute lymphocytic leukemia (B-ALL), non-Hodgkin lymphoma (NHL), and multiple myeloma (MM) (1–5). Currently, CD19 and BCMA are the most common target antigens for CAR-T cell therapy (6). To date, there are six CAR-T cell products approved by the US Food and Drug Administration (FDA) for the treatment of R/R B cell malignancies, including four products targeting CD19 and two products targeting B cell maturation antigen (BCMA) (7, 8). To improve the efficacy of CAR-T cell therapy, in addition to the optimization of the structure and infusion dose of CAR-T cells, patient preparation prior to CAR-T cell infusion is also crucial, such as bridging therapies and lymphodepletion chemotherapy (9). Bridging therapies generally refer to the therapies which are performed during CAR-T cell manufacturing, and they play an essential role in preventing disease progression and reducing tumor burden. In addition, the decreased tumor burden could reduce the risk of severe cytokine release syndrome (CRS). Due to individual differences and different clinical experience between centers, bridging strategies usually vary between patients. At present, it has been demonstrated that chemotherapies, radiotherapy, immunotherapies, and autologous stem cell transplantation (ASCT) could serve as effective bridging therapies prior to CAR-T cell infusion (6, 9–14). Nevertheless, there are still unmet needs for bridging therapies for those patients who have experienced disease progression after multiline chemotherapies and radiotherapy or present with bulky disease (maximal tumor diameter ≥ 7.5 cm).

Cryoablation is a minimally invasive therapy which has been successfully utilized for the treatment of multiple solid tumors, such as liver cancer, lung cancer, breast cancer, and prostate cancer. It exhibits several unique advantages, such as rapidly achieving local tumor control, palliating pain, maintaining local tissue integrity, and monitoring the treatment area in real time with computed tomography (CT) or magnetic resonance imaging (MRI). There are slight differences of cryoablation application in different tumor types. In general, it could achieve local radical treatment of tumors < 3 cm in diameter, and it could rapidly reduce local tumor burden for primary or metastatic tumors > 3 cm in diameter. The mechanisms of cryoablation-mediated tumor killing are complex. Similar to radiotherapy, cryoablation could induce tumor necrosis and promote the release of tumor antigens and damage-associated molecular patterns (DAMPs), such as high mobility group protein 1 (HMGB1) and heat shock proteins (HSPs), as well as tumor antigen cross-presentation, eventually triggering systemic anti-tumor immune responses through activating endogenous innate immune cells and increasing the infiltration of tumor-specific T cells at both primary tumor and metastatic sites (15–17). In addition, several studies have demonstrated that cryoablation could also induce the elimination of distant metastases, which is known as the abscopal effect (16). However, cryoablation-mediated anti-tumor immune responses are usually insufficient to overcome tumor immune escape and trigger the abscopal effect. Recently, numerous studies have demonstrated that cryoablation combined with immunotherapies, such as PD-1 blockade, CTLA-4 blockade, adoptive dendritic cells (DCs), and natural killer cells (NKs), could induce synergetic anti-tumor effects as well as the abscopal effect through activating robust systemic anti-tumor immune responses (18–22). However, combination therapy with CAR-T cell therapy and cryoablation has not yet been reported. To rapidly achieve local tumor control and reduce tumor burden during CAR-T cell manufacturing, we explored the safety and efficacy of this novel combination therapy in our present study.

## 2 Case presentation

**Figure 1:**
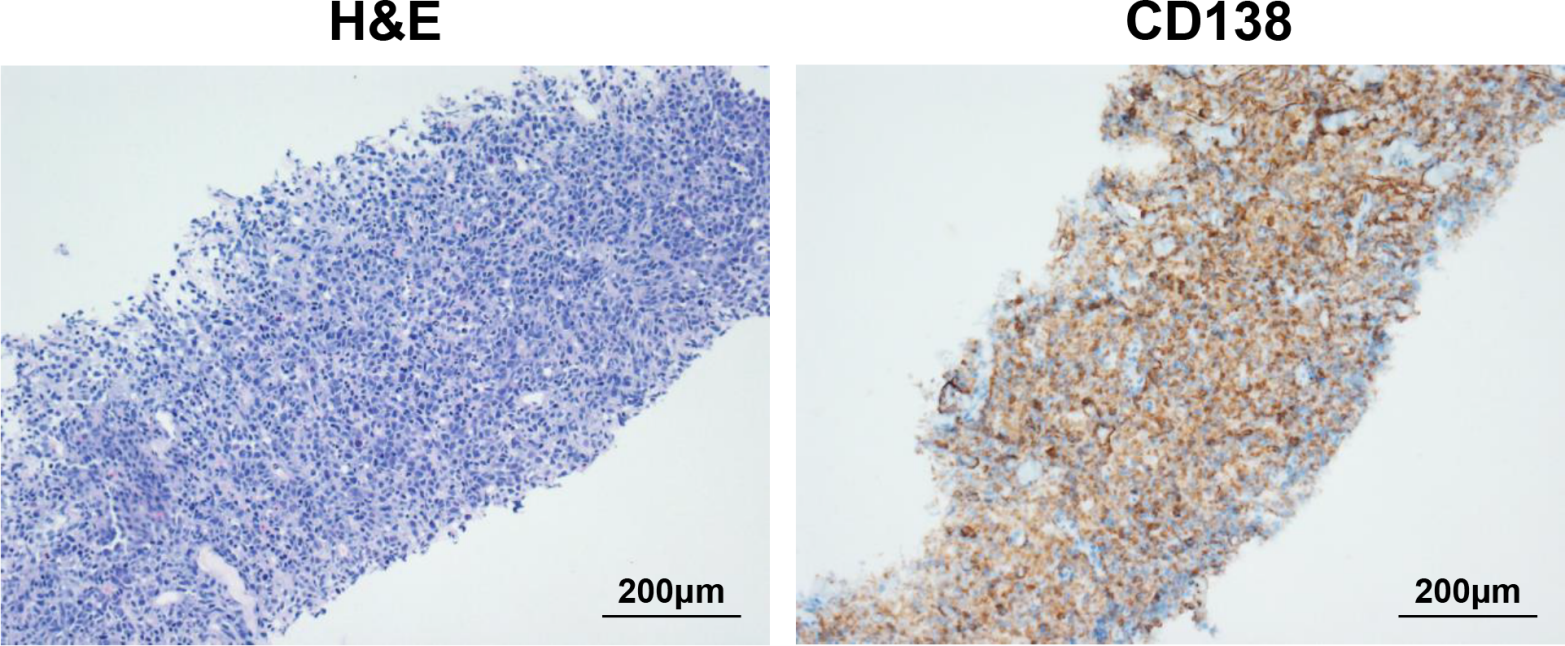
Biopsy of neck mass showing extramedullary disease (EMD). On the left is hematoxylin and eosin (H&E) staining of neck mass (original magnification 200×), and on the right is immunohistochemical (IHC) staining for CD138 (original magnification 200×).

**Figure 2:**
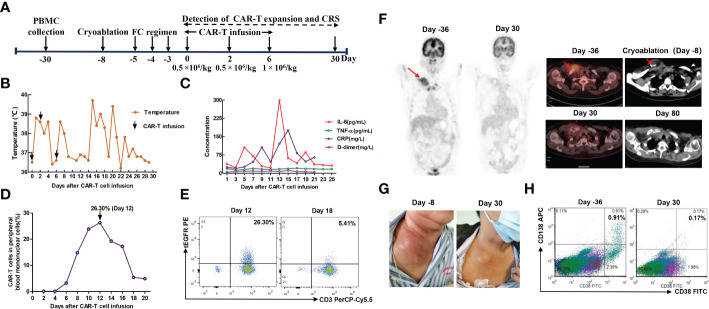
**(A)** Timeline of patient preparation and CAR-T therapy for patient 1 from peripheral blood collection to 1 month after anti-BCMA CAR-T cell infusion, including bridging cryoablation, lymphodepleting chemotherapy, and CAR-T infusion. **(B)** Changes in body temperature after CAR-T cell infusion. **(C)** The levels of IL-6, TNF-α, C-reactive protein and D-dimer in serum at different time points after CAR-T cell infusion. **(D, E)** The percentage of anti-BCMA CAR-T cells in peripheral blood mononuclear cells (PBMCs) at different time points. The plot was gated on PBMCs. **(F)** CT-guided percutaneous cryoablation rapidly induced liquefaction necrosis of the neck mass. PET/CT showed that the neck mass was eliminated 1 month after combination therapy with cryoablation and CAR-T therapy, and it remained in remission 80 days after CAR-T cell infusion. **(G)** The neck mass showed a marked reduction in size 1 month after CAR-T cell infusion. **(H)** Flow cytometry analyses showed that MM cells was remarkably decreased in bone marrow 1 month after CAR-T cell infusion. The plot was gated on CD19 negative, CD45 dim or negative and/or CD56 positive cells.

The first patient was a 65-year-old female who was admitted to hospital due to low back pain 1 year ago and was diagnosed with MM by positron emission tomography/computed tomography (PET/CT) and bone marrow biopsy. Chest and abdominal CT showed multiple bone destruction and hilar and mediastinal lymphadenopathy. Then she was treated with 2 cycles of PAD regimen (bortezomib, adriamycin, and dexamethasone). Unfortunately, a bulky subcutaneous mass appeared on the right side of her neck 3 months after initial diagnosis, and fine-needle aspiration biopsy suggested extramedullary disease (EMD) (**Figure 1**). Due to disease progression, the chemotherapy regimen was adjusted to bendamustine plus VTD regimen (bendamustine, bortezomib, thalidomide, and dexamethasone) (**Table 1**). However, the neck mass was still enlarged, the size of which rapidly increased to 2.4 × 7.8 × 8.2 cm. Given that the patient gained limited benefits from conventional chemotherapies, she was enrolled in a clinical trial of anti-BCMA CAR-T cell therapy (ChiCTR2100046014). These anti-BCMA CAR-T cells were constructed with a mouse single-chain variable fragment (scFv). In order to prevent the progression of the neck mass and reduce tumor burden during CAR-T cell manufacturing, radiotherapy was recommended. Nevertheless, the patient refused radiotherapy. After multidisciplinary assessment by oncologists, radiologists, and interventional radiologists, the patient was eligible for cryoablation. Thus, she received CT-guided percutaneous cryoablation 1 week before anti-BCMA CAR-T cell infusion (**Figure 2A**). The cryoablation was performed using a Galil Medical system and percutaneous cryoprobes. According to pretreatment CT images, three cryoprobes were placed to entirely cover the neck mass. Two freeze-thaw cycles were performed throughout the ablation process, with each cycle of freezing 15 minutes and thawing 5 minutes, and the ablation area was monitored by CT in real time. The warm pads were used to minimize freezing-induced skin injury. Except for minor bleeding, no significant adverse events were observed. Then she was preconditioned with FC regimen (fludarabine 25 mg/m^2^ day -5 to day -3, cyclophosphamide 250 mg/m^2^ day -5 to day -3). In order to prevent severe CRS, the patient was administrated with anti-BCMA CAR-T cells in a dose-escalation scheme with an initial dose of 0.5 × 10^6^ cells/kg, followed by 0.5  × 10^6^ cells/kg and 1 × 10^6^ cells/kg, respectively (**Figure 2A**).The next day after the first dose of CAR-T cells, the patient vomited and had a fever with a temperature of 38.8°C (**Figure 2B**), which suggested grade 1 CRS. Thus, supportive care was provided. The peak of CAR-T cell levels in peripheral blood mononuclear cells (PBMCs) reached 26.3% on day 12 (**Figure 2D, E**). Serum IL-6 reached peak levels of 298.7 pg/mL 13 days after CAR-T cell infusion (**Figure 2C**). One month after anti-BCMA CAR-T cell therapy, the neck mass was significantly decreased in size (**Figure 2G**) and PET/CT showed the elimination of EMD (**Figure 2F**). In addition, the pathological examination by percutaneous image-guided biopsy revealed the necrosis of neck mass 1 month after CAR-T cell therapy (**Supplementary Figure S1**), and flow cytometry showed that MM cells in bone marrow was decreased (**Figure 2H**). However, the serum immunofixation electrophoresis remained positive. After the comprehensive assessment, the patient achieved a VGPR 1 month after CAR-T cell infusion. EMD remained in remission 80 days after CAR-T cell infusion (**Figure 2F**). Unfortunately, pleural invasion occurred and massive pleural effusion was generated 6 months after CAR-T cell infusion, and the patient died of disease progression.

**Table 1:** The general clinical characteristics of the patients.

Patient information	Patient 1	Patient 2
**Age**	65	70
**Sex**	female	male
**Diagnosis**	MM IgD (II stage, Lambda Light chain)	PCLBCL-LT (T2 stage)
**Autologous stem cell transplantation**	No	No
**Bulky disease**	neck mass, 2.4 × 7.8 × 8.2 cm in size	thigh mass, 8.5 × 7.3 × 9.3 cm in size
**Time of initial diagnosis**	9 months ago	1 year ago
**Prior therapies**	PAD (bortezomib, adriamycin, and dexamethasone) and bendamustine plus VTD (bortezomib, thalidomide, and dexamethasone)	R-CHOP (rituximab, cyclophosphamide, doxorubicin, vincristine, and prednisone), R-DICE (rituximab, dexamethasone, ifosfamide, cisplatin, and etoposide), P-GEMOX (pegaspargase, gemcitabine, and oxaliplatin), and radiotherapy

The ISS stage was performed according to the International Myeloma Working Group (23), the staging of lymphomas was performed according to NCCN guidelines Version 4.2022: B-Cell Lymphomas.

**Figure 3:**
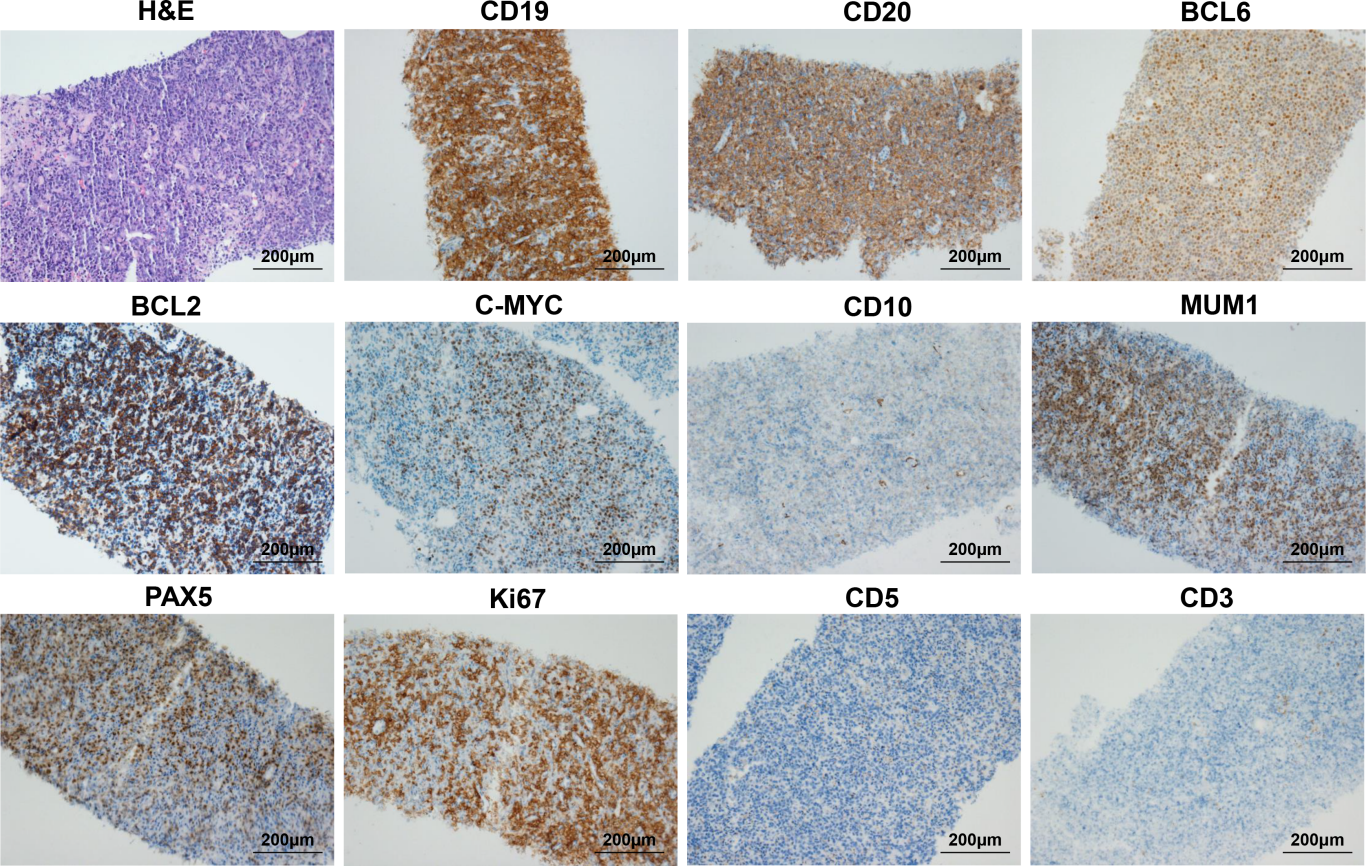
Biopsy of right thigh mass showing primary cutaneous diffuse large B cell lymphoma, leg type (PCLBCL-LT) with c-MYC/BCL2 double-expressor. The first image is H&E staining of thigh mass (original magnification 200×), and the other images are IHC staining for CD19, CD20, BCL6, BCL2, C-MYC, CD10, MUM1, PAX5, Ki-67, CD5, and CD3 (original magnification: 200×).

**Figure 4:**
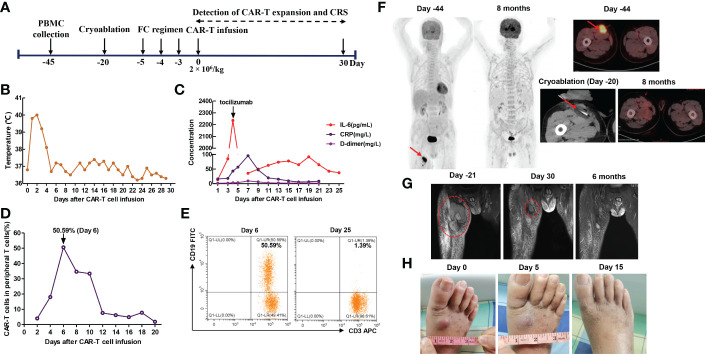
**(A)** Timeline of patient preparation and CAR-T therapy for patient 2 from peripheral blood collection to 1 month after anti-CD19 CAR-T cell infusion, including bridging cryoablation, lymphodepleting chemotherapy, and CAR-T cell infusion. **(B)** Changes in body temperature after CAR-T cell infusion. **(C)** The levels of IL-6, C-reactive protein and D-dimer in serum at different time points after CAR-T cell infusion. **(D, E)** The percentage of anti-CD19 CAR-T cells in peripheral T cells at different time points. The plot was gated on CD3+ cells (T cells). **(F)** CT-guided percutaneous cryoablation rapidly induced liquefaction necrosis of the bulky thigh mass, PET/CT showed that the right thigh mass remained in remission 8 months after CAR-T cell infusion. **(G)** MRI revealed that the thigh mass was remarkably decreased 1 month after the combination of cryoablation and CAR-T therapy and it was completely eliminated 6 months after CAR-T cell infusion. **(H)** Combination therapy induced the remission of newly emerging distant metastases 15 days after CAR-T cell infusion.

The second patient was a 70-year-old man who presented with a subcutaneous mass in the right anterior thigh and was diagnosed with primary cutaneous diffuse large B cell lymphoma, leg type (PCLBCL-LT) with c-MYC/BCL2 double-expressor through fine needle aspiration biopsy 1 year ago. Immunohistochemistry analysis showed CD19 (+), CD20 (+), BCL2 (+), C-MYC (+), BCL6 (+), MUM1 (+), PAX5 (+), Ki-67 (+, 80%), CD10 (-), CD3 (-), and CD5 (-) (**Figure 3**). In situ hybridization for BCL2, BCL6 and C-MYC BCL2 and C-MYC was negative. The patient underwent multi-line chemotherapies, including R-CHOP (rituximab, cyclophosphamide, doxorubicin, vincristine, and prednisone), R-DICE (rituximab, dexamethasone, ifosfamide, cisplatin, and etoposide), and P-GEMOX (pegaspargase, gemcitabine and oxaliplatin). However, the thigh mass was still enlarged. Thus, he received radiotherapy, and the volume of thigh mass was gradually decreased. Unfortunately, the thigh mass was enlarged again 3 months after radiotherapy (**Table 1**). Thus, he requested anti-CD19 CAR-T cell therapy. MRI showed the right thigh mass was about 8.5 × 7.3 × 9.3 cm in size 21 days before anti-CD19 CAR-T cell infusion (**Figure 4G**), which suggested tumor progression. Therefore, he immediately received CT-guided percutaneous cryoablation to control disease progression and reduce tumor burden after an evaluation by a multidisciplinary team (**Figure 4A**). The cryoablation was performed with a Galil Medical system. Four cryoprobes were placed to entirely cover the larger thigh mass under CT guidance, and two freeze-thaw cycles were performed, with each cycle of freezing 15 minutes and thawing 5 minutes. No serious adverse events occurred throughout the ablation process. Due to the absorption of necrotic tumor tissues, the right thigh circumference was decreased from 52.6 cm to 48.4 cm 15 days after cryoablation. After lymphodepletion chemotherapy with FC regimen, he was treated with the commercial anti-CD19 CAR-T cell product (axicabtagene ciloleucel) at a recommended dose of 2 × 10^6^ cells/kg. Within 4 days after CAR-T cell infusion, he presented with hypotension and recurrent fever (**Figure 4B**), and serum IL-6 reached its peak level of 2235.78 pg/mL 4 days after CAR-T cell infusion, which suggested severe CRS (**Figure 4C**). Thus, norepinephrine was utilized to elevate blood pressure. To attenuate excessive inflammatory responses, tocilizumab was administered at a dose of 8 mg/kg. CAR-T cell expansion in peripheral T cells reached peak levels of 50.59 % on day 6 (**Figure 4D, E**), which was gradually decreased during the subsequent 2 weeks. MRI showed that the thigh mass was remarkably decreased 30 days after CAR-T cell infusion (**Figure 4G**). Intriguingly, a newly emerging mass on the dorsum of the right foot during CAR-T cell manufacturing was completely eliminated 15 days after CAR-T cell infusion (**Figure 4H**). In addition, the right thigh circumference was decreased from 48.4 cm to 42.5 cm 1 month after CAR-T cell infusion. After clinical assessment, he achieved PR 1 month after CAR-T cell infusion. Interestingly, the bulky disease was completely eliminated six months after CAR-T cell infusion (**Figure 4G**). To date, the patient still remained in remission (**Figure 4F**).

## 3 Methods

### 3.1 Histopathological examination and immunohistochemistry

For histopathological examination, biopsy specimens were fixed in 4% paraformaldehyde for 48 hours and then embedded in paraffin. Sections were cut in 4 μm of thickness and used for hematoxylin and eosin (H&E) staining. For immunochemical analysis, paraffin sections were deparaffinized with xylene and rehydrated through graded alcohols. Then, sections were boiled in sodium citrate to repair antigen, and endogenous peroxidase enzyme was inactivated using 3% H_2_O_2_ solution for 10 min. After blocking with 5% milk, sections were incubated with the primary antibody overnight at 4°C and then incubated with a biotinylated secondary antibody for 2 h at room temperature. Immunocytochemical (IHC) staining was performed using primary antibodies against CD138 (Mouse, 1:200, Cell Signaling Technology) on paraffin-embedded neck mass. IHC staining was performed using primary antibodies against CD19, CD20, BCL6, BCL2, C-MYC, CD10, MUM1, PAX5, Ki-67, CD5, and CD3 (Mouse, 1:200, Cell Signaling Technology) on paraffin-embedded thigh mass, respectively. Afterwards, these sections were stained with a DAB chromogenic solution for 5 to10 minutes and counterstained with hematoxylin.

### 3.2 Flow cytometry

To detect anti-BCMA CAR-T cell expansion in peripheral blood, the following antibodies were used: CD45-Alexa Fluor 488, CD3-PerCP5.5, and tEGFR-PE (BD Biosciences, San Diego, USA). CD45/SSC gating was applied to identify PBMCs and further check for anti-BCMA cells. Because these anti-BCMA CAR-T cells were constructed with CAR gene integrated with truncated human epidermal growth factor receptor (tEGFR), they could be directly detected using CD3-PerCP5.5 and tEGFR-PE antibodies in the present study. To detect MM cells in bone marrow, the following antibodies were utilized: CD45-V500, CD19-PE-Cy7, CD20-PerCP-Cy5.5, CD56-APC-H7, CD38-FITC, and CD138-APC (BD Biosciences, San Diego, USA). MM cells were identified based on CD138 positivity and strong CD38 expression. To detect anti-CD19 CAR-T cell expansion in peripheral blood, CD3-APC and CD19-FITC antibodies (BD Biosciences, San Diego, USA) were used. In addition, debris was first excluded based on light scatter properties in the above flow cytometry analysis.

### 3.3 Cytokine measurement

Serum levels of TNF-α, IL-6, CRP were detected by commercial ELISA kits (R&D Systems, Minneapolis, USA) and the protocols were adopted according to manufacturer’s instructions, and serum D-dimer levels were measured by commercial D- dimer assay kit (Diagnostica Stago, Asnieres, France).

## 4 Discussion

Despite the great success of CAR-T cell therapy in R/R B cell malignancies in recent years, there are still numerous unmet needs that remain to be addressed. For example, there are no unified standards for the application of bridging therapies. In fact, many candidates for CAR-T cell therapy experience disease progression during CAR-T cell manufacturing. Therefore, effective bridging therapies prior to CAR-T cell therapy are urgently needed to control disease progression (9). Case 1 was a MM patient with EMD, who was resistant to bortezomib-based chemotherapy regimens. Due to high tumor burden, MM patients with EMD usually gain limited benefits from conventional chemotherapies and have a poor prognosis. Thus, patient 1 was enrolled in the anti-BCMA CAR-T cell clinical trial. Unfortunately, the neck mass was rapidly enlarged during CAR-T cell manufacturing. Case 2 was an elderly PCDLBCL-LT patient with MYC/BCL2 double-expressor, and presented with a bulky thigh mass. PCLBCL-LT is a relatively rare and aggressive subtype of NHL with an unfavorable prognosis (24). Similar to DLBCL, R-CHOP regimen remains to be the recommended first-line therapy for PCDLBCL-LT. However, patient 2 was also resistant to radiotherapy and multiple chemotherapy regimens, including R-CHOP, R-DICE, P-GEMOX. Thus, he requested anti-CD19 CAR-T cell therapy. However, the bulky thigh mass was an intractable problem. Clinically, EMD and extranodal involvements are uncommon. In particular, patients with bulky disease were frequently excluded from CAR-T cell clinical trials (25). Consequently, due to the lack of clinical trials specially designed for the patients with bulky disease, there is no consensus on patient preparation for CAR-T cell therapy in these individuals. In addition, it’s reported that EMD is associated with the risk of higher grade of CRS and CAR-T-cell-related encephalopathy syndrome (26, 27). To control disease progression and reduce the risk of severe CRS in these two patients, bridging therapies are urgently needed. To date, radiotherapy has been demonstrated to be an effective bridging therapy for local tumor control during CAR-T cell manufacturing (12, 13). Unfortunately, the patients refused or were resistant to radiotherapy in the present study. In addition, the relatively longer course of radiotherapy limits its efficacy in rapidly progressive tumors.

Cryoablation is a minimally invasive therapy with the advantage of rapid local tumor control and symptom palliation under the real-time monitoring of imaging systems (28), and has been widely utilized in multiple solid tumors in recent years, especially for those who couldn’t tolerate or refuse surgery and radiotherapy. In general, 2 or 3 freeze-thaw cycles are performed throughout the ablation process, with each cycle of freezing 15 minutes and thawing approximately 5 minutes. The number of cryoprobes utilized in cryoablation mainly depends on the size, shape and site of tumor. Cryoablation usually utilizes liquefied gases, such as argon and helium. The rapid expansion of high-pressure argon results in the temperature of the distal end of cryoprobes as low as -170 °C, while the expansion of high-pressure helium makes the temperature of the distal end of cryoprobes rapidly rise to 40 °C. Tumor destruction mediated by cryoablation involves multiple mechanisms, such as direct induction of cell death, vascular disruption and ischemia, and the activation of immune responses (15). In addition, it is an effective salvage therapy for patients who have experienced local recurrence after radiotherapy (29). Therefore, CT-guided percutaneous cryoablation was performed to rapidly control local disease progression after multidisciplinary assessment in our study. Due to large tumor size, four cryoprobes were utilized throughout the ablation process in patient 2. It rapidly induced liquefaction necrosis of these local tumors (**Figure 2F**, **Figure 4F**), and the right thigh circumference of patient 2 was remarkably decreased 2 weeks after cryoablation. Subsequently, they received CAR-T cell therapy and had a superior response 1 month after CAR-T cell infusion. To prevent severe CRS, body temperature, the levels of several vital cytokines, the persistence of CAR-T cells were detected within 1 month after CAR-T cell infusion, and all adverse events were controllable. In the present study, the interval between cryoablation and CAR-T cell infusion depended on the rate of local disease progression during CAR-T cell manufacturing. To the best of our knowledge, this is the first study to assess the safety and efficacy of combination therapy with cryoablation and CAR-T cell therapy. Patient 2 remained in remission 8 months after CAR-T cell infusion. Unfortunately, patient 1 developed pleural invasion 6 months after anti-BCMA CAR-T cell infusion and died of disease progression, which may be partly attributable to the decreased persistence of CAR-T cells constructed with a mouse scFv.

As an effective local ablative therapy, cryoablation could induce strong immunogenicity (30), and cryoablation-related adverse events are usually mild, such as bleeding and skin damage. The repeated freeze-thaw cycles rapidly induce tumor cell necrosis and apoptosis and microvascular destruction. The sharply demarcated liquefaction necrosis is observed at the center of the ablative lesions under the real-time monitoring of CT, whereas apoptosis is mediated by sublethal cold temperatures in the peripheral zone (15). Tumor cell necrosis results in the release of DAMPs and hidden tumor antigens, thereby simultaneously activating endogenous innate immune responses and tumor-specific immune responses, and it could also sensitize tumors to immunotherapy and contribute to synergistic anti-tumor effects as well as the abscopal effect to prevent tumor metastasis and recurrence (9–16). Intriguingly, the abscopal effect was also observed in our study (**Figure 4H**). In addition, several studies have demonstrated that cryoablation could increase TCR diversity and promote the expansion of TCR clones in tumor tissues, especially the expansion of anti-tumor CD8 + T cells (17, 31). Notably, there are several factors which could influence the efficacy of cryoablation, such as the number and positioning of cryoprobes and the number of freeze-thaw cycles. Therefore, if cryoablation is adopted as a bridging therapy for local tumor control prior to CAR-T cell infusion, it should be performed by trained teams after a careful evaluation by a multidisciplinary team.

## 5 Conclusion

In conclusion, our study demonstrated the satisfactory safety and efficacy of combination therapy with cryoablation and CAR-T cell therapy for the first time, and it indicates that cryoablation might be a novel bridging therapy for local tumor control during CAR-T cell manufacturing. In addition, it also expands the application of cryoablation. Besides solid tumors, cryoablation could also be applied for local tumor control in hematological malignancies. Given the small sample size of our study and the immunogenic effects of cryoablation in different tumor types, the safety and efficacy of this novel combination therapy as well as the optimal time points for bridging cryoablation need to be further confirmed in large-scale prospective studies.

## Data availability statement

The original contributions presented in the study are included in the article/**Supplementary Material**. Further inquiries can be directed to the corresponding author.

## Ethics statement

The studies involving human participants were reviewed and approved by Ethics Committee of Jinshazhou Hospital, Guangzhou University of Chinese Medicine. The patients/participants provided their written informed consent to participate in this study. Written informed consent was obtained from the individual(s) for the publication of any potentially identifiable images or data included in this article.

## Author contributions

YX, LQ and RL devised this combination therapy. XZ, CC and HZ analyzed the data and wrote the manuscript. XZ, JW, and LC took care of the patients and collected the data. YX and XZ revised the manuscript. All authors contributed to the article and approved the submitted version.
