# Supplementary material for: Case report: Cryoablation as a novel bridging strategy prior to CAR-T cell therapy for B cell malignancies with bulky disease
Source: Front Oncol. 2023 Jan 27;13:1008828. doi: 10.3389/fonc.2023.1008828 (PMC9911860; doi:10.3389/fonc.2023.1008828)
Supplement: Supplementary file 1 [file Image_1.pdf]

Supplementary materials

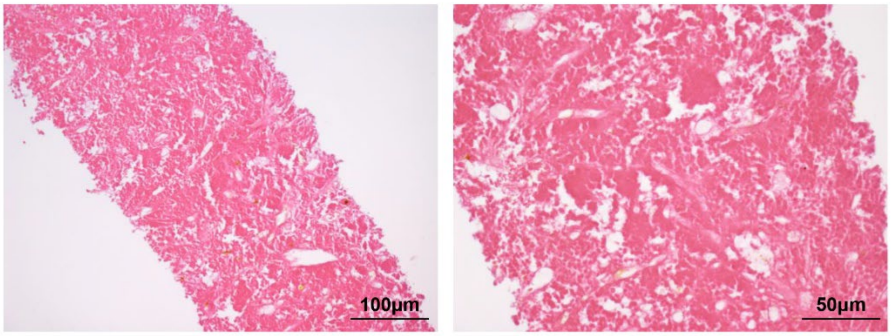

Figure S1 Hematoxylin and eosin staining of neck mass (original magnification: 100× and 200×). The pathological examination revealed the necrosis of neck mass 1 month after CAR-T cell therapy combined with cryoablation.
